# Bogus Veterinary Medical Degrees

**Published:** 1881-10

**Authors:** 


### EDITORIAL DEPARTMENT.

### BOGUS VETERINARY MEDICAL DEGREES.

IN all the discussion over bogus medical diplomas which has been going on during the past year nothing has been said about bogus veterinary degrees. Yet from information kindly furnished us by Prof. E. S. Bates, Dean of the Columbia Veterinary College and School of Comparative Medicine, we infer that considerable business in this direction has been done somewhere in this country.

Immediately after the organization of the College aforesaid, some four or five years ago, requests for the price of a diploma began to come in. For two or three years letters offering various sums for the privilege of a veterinary degree were received, at the rate of one or two a month. Of late, we are told, this department of correspondence is less active. The letters in question came from every quarter, including England and India. The majority, however, came from the West. The price offered varied from a shake of the hand to five hundred dollars.

We print, in part, one characteristic letter from a Western man who "meant business":

"Desire a diploma, but cannot spend time necessary to get one. Will give just one hundred and fifty dollars for the same—if we can deal; if not, all right. Anything you may say upon the subject will be strictly between us.

"Sincerely, etc.

"P. S.—You need not take the trouble to send me a lecture upon the immorality of my proposition; it would be wasted Am a Western man, and will do just as I agree, quietly, as it will be of service to me and cost you nothing."

Another letter, a portion of which we print *verbatim*, has some striking literary merits. There is a pleasing candor, also, about the writer's proposition which goes to make us wish he had succeeded better in his request :

"DER SIR.— * * * * I want you to do me a faver if you Can posable & I will Reward you Sure when I Come to the College I have Study vet Book for A long time & done good deal of doctern & had good Results in all my practis But when I go to Collect my Bills grate meny of our people will Say you haven not got no diaploma & I wont pay you tel you git one So if you Can assit eny way pleas do it if you Can send me A diaploma by express Cod then I Can Collect wat is due to me & will Beable to Come to See you and give you a harty Shake hands I am poore & ned assistence I am young only 28 year old if this wont do Could I be examin by letter & git one pleas give me the best information you Can in Regard to it pleas answer Soon I want to do all I Can So I Can Come out to new york nex fall I think I Can bring one or to more Students when I Come.

"Yours truly."

With such a demand for veterinary degrees, it seems hardly possible to believe that there is not some institution which supplies them. Of this, however, we have no certain knowledge. The notorious Buchanan had no veterinary department in his University, which shows some lack of enterprise and knowledge on the part of that eminent man.

It is very well known that there is a veterinary society in this county which disposes of licenses or diplomas at rates which amply compensate those engaged in the business. No society, however, can give a degree, and a license only entitles a man to use the letters V. S.

It seems to have been through a great oversight that one of the two American Veterinary Colleges gives to its alumni only the title "Veterinary Surgeon." Its degree is really, therefore, only a license, and its alumni are likely to be confounded with those who assume the letters V. S., or with those who have bought it from a chartered veterinary society. It should in justice to its graduates, as well as for the better standing of the profession, give a regular doctorate. We should be glad to see the change made in response to our suggestion. At present the Columbia Veterinary College is the only institution in the United States which gives the degree of Doctor of Veterinary Science.

